# Tomato Aphid (*Aphis gossypii*) Secreted Saliva Can Enhance Aphid Resistance by Upregulating Signaling Molecules in Tomato (*Solanum lycopersicum*)

**DOI:** 10.3390/ijms241612768

**Published:** 2023-08-14

**Authors:** Khadija Javed, Yong Wang, Humayun Javed, Chen Wang, Chuang Liu, Yuqian Huang

**Affiliations:** 1 Plant Protection College, Shenyang Agricultural University, No. 120 Dongling Road, Shen He District, Shenyang 110866, China; khadijajaved829@gmail.com (K.J.);; 2 Department of Plant Pathology, Agriculture College, Guizhou University, Guiyang 550025, China; yongwangbis@aliyun.com; 3 Rothamsted Research West Common Harpenden, Hertfordshire AL5 2JQ, UK; hjhumayun@gmail.com

**Keywords:** *Aphis gossypii*, signaling, jasmonic acid (JA), salicylic acid (SA), infiltration of the saliva, defense mechanism, *A. gossypii* performance, choice partiality, feeding performance

## Abstract

This study investigated the impact of *Aphis gossypii* watery saliva on the induction of tomato (*Solanum lycopersicum*) plant resistance. To examine the role of *A. gossypii* saliva, we collected watery saliva from *A. gossypii* after a 48 h feeding period on an artificial diet. SDS-PAGE resolving gel 12% was used to separate the salivary proteins. Relative expression of gene analysis revealed that the intrusion of *A. gossypii* saliva dripping onto *S. lycopersicum* leaves triggered robust defense responses mediated by a signaling molecule, i.e., salicylic acid, while the signaling molecule’s jasmonic acid-dependent defense responses were moderately activated. Aphid saliva infiltrated *S. lycopersicum* leaves slowed the intrinsic rate of population growth of *A. gossypii* and significantly reduced the number of nymphs produced daily, compared to untreated leaves. During a choice test with untreated *S. lycopersicum*, aphids showed a repellent response towards saliva-infiltrated *S. lycopersicum*. Moreover, the (EPG) electrical penetration graph analysis demonstrated that the eating pattern of *A. gossypii* compared to untreated *S. lycopersicum*, that had been exposed to saliva was negatively impacted. These results provide compelling evidence for the involvement of salivary components of *A. gossypii* in inducing resistance against aphids in *S. lycopersicum* plants. Furthermore, the study underscores the crucial role of watery saliva in the intricate interactions between aphids and plants. The activation of pathways was also part of the defensive response (jasmonic acid (JA), salicylic acid (SA) signaling molecules). The findings of this research deliver valuable insights into the potential of watery aphid saliva as a natural defense mechanism against aphid infestations in *S. lycopersicum* crops.

## 1. Introduction

Over the course of their evolutionary history, plants have developed a variety of defense mechanisms in response to attacks from insects. These defense mechanisms can be categorized into direct defenses and indirect defenses. Insect development is stymied by direct defenses such as the creation of proteinase inhibitors (PIs) and other lethal secondary metabolites. To counteract herbivores or attract predators, plants use indirect defenses like emitting volatile chemicals. Plants respond to herbivory by exuding these volatile chemicals [1]. Two key signaling molecules, jasmonic acid (JA) and salicylic acid (SA), play significant roles in triggering plant defense responses, JA and SA are involved in the induction of different defense pathways [2]. According to current theories, plants activate the JA-mediated defense pathway when they are infested by necrotrophic pathogens or subjected to leaf-chewing herbivores. Conversely, Biotrophic pathogens and phloem feeders are the primary stimuli for SA-dependent defenses [3]. Aphids, belonging to the Hemiptera: Aphididae group, are significant pests that feed on the phloem of plants. Their infestations have considerable economic implications, leading to substantial agricultural and horticultural losses worldwide [4]. In numerous aphid–plant interactions, the activation of a defense pathway dependent on salicylic acid (SA) has been well-documented in response to aphid feeding. For instance, the green peach aphid (*Myzus persicae*) has been observed to induce the SA-dependent defense pathway in tomato, tobacco, and *Arabidopsis* plants, ref. [5] while the Russian wheat aphid (*Diuraphis noxia*) triggers a similar response in wheat [6]. Genes involved in the jasmonic acid (JA) signaling pathway, including lipoxygenase (*LOX*) and proteinase inhibitors (PIs), are upregulated when the potato aphid *Macrosiphum euphorbiae* feeds on tomato plants. These JA-related genes are active in both compatible and incompatible aphid-to-tomato plant interactions [7]. Similar to this, the sorghum-eating green bug aphid *S. graminum* [8] *M. persicae* feeding on *Arabidopsis* has been shown to induce JA-related genes [9].

Plants possess the remarkable ability to detect specific chemical signals present in the saliva of herbivores, which serve as cues for potential threats. These signals, known as herbivore-associated elicitors or herbivore-associated molecular patterns (HAMPs), play a crucial role in activating targeted defense responses within plants, while minimizing the associated costs to their overall fitness [10]. These compounds have been shown to trigger the activation of various defense responses in plants, including the production of salicylic acid (SA), jasmonic acid (JA), ethylene (ET), and reactive oxygen species (ROS), all of which are vital for effective defense against herbivory [11]. During the probing and feeding process, aphids employ various defensive mechanisms. At first, they protect their stylets from harm by secreting gelling saliva, which can harden into a tube-like sheath [12]. Aphids inject apoplasts and plant cells with watery saliva, a more nuanced blend of enzymes and defensive components [13]. According to the gene-for-gene model theory, plant resistance (R) proteins detect certain components or elicitors found in aphid saliva. These R proteins contain a nucleotide binding site–leucine rich repeat (NBS-LRR). This recognition can trigger a defense response, leading to resistance against aphids [14]. The role of watery saliva in plant defense against aphids has been thoroughly investigated in research studies, especially in *M. persicae*. Watery saliva’s evocative power has been well-established., despite the fact that particular aphid elicitors have not yet been discovered. In experiments where salivary components from *M. persicae*, ranging in size from 3–10 kDa, were infiltrated, protection to aphids incorporated into *Arabidopsis* plants was activated, leading to a reduction in their ability to reproduce. Furthermore, this infiltration resulted in the activation of 52 genes associated with stress responses, including senescence-associated protein 1 and cytochrome P450, indicating their involvement in plant defense mechanisms upon aphid feeding [15]. Different hydrolytic and oxidative enzymes found in aphid saliva, like pectinases and polyphenol oxidase (PPO), have been demonstrated to stimulate plant defense responses [16]. Additionally, two potential *M. persicae* salivary elicitors, namely Mp10 and Mp42, have been identified. When these elicitors were overexpressed, aphid fecundity decreased. In *Nicotiana benthamiana*, Mp10 specifically led to chlorosis and initiated the signaling pathways involving salicylic acid (SA) and jasmonic acid (JA), emphasizing the significance of both of these pathways in activating plant defense mechanisms [17].

*Aphis gossypii*, is a highly prevalent and destructive pest that inflicts significant damage to tomato crops worldwide. This pest not only directly feeds on the phloem sap of tomato but also acts as a vector for transmitting viruses [18]. The feeding activity of *A. gossypii* has been observed to increase the *LOX*, phenylalanine ammonia lyase (*PAL*), and PPO enzyme activity in tomato. These enzymes are associated with both the JA and SA pathways. Allene oxide synthase (*AOS*) and phenylalanine ammonia-lyase (*PAL*), two enzymes involved in the production of JA and SA, both had increased mRNA levels [19]. While several proteins have been found in *A. gossypii’s* watery saliva [20], it is still unclear what role, if any, saliva plays in aphid–tomato interactions. We collected the watery saliva of *A. gossypii* and infiltrated it into the leaves of *S. lycopersicum* to determine whether or not the saliva of aphids has an effect on the activation of defense mechanisms. To evaluate the effect of saliva on the defense response of *S. lycopersicum* and the subsequent performance of the aphids, we used RT-qPCR, aphid bioassays, and EPG recording techniques.

## 2. Results

### 2.1. Local Defence Responses Were Triggered by A. gossypii Feeding

**Figure 1:**
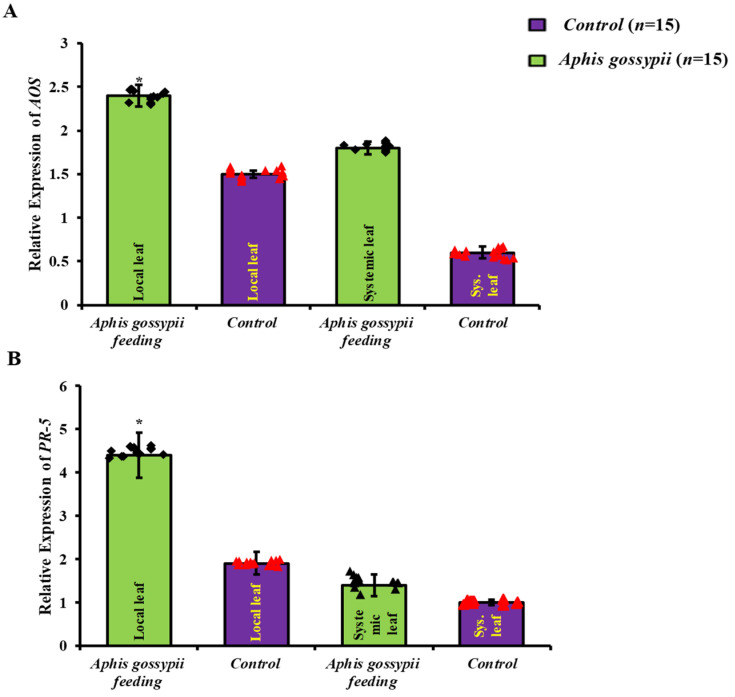
Genes implicated in the JA (**A**) and SA (**B**) defensive responses in *S. lycopersicum* leaves, and their relative expression after 48 h of aphid feeding. Mean ± SE was the format used to present the data. The green and purple bars depict the local and systemic leaves, respectively. Statistically significant differences between groups are indicated by a “*” (*p* < 0.05, *t*-test, *n* = 15).

To examine if aphid infestation might improve resistance in *S. lycopersicum*, we found two genes connected with JA- and SA-mediated defensive mechanisms, which exhibited differences in expression while being fed upon by *A. gossypii* (Figure 1A,B). A considerable increase in the relative expression of the JA-responsive gene *AOS* in local leaf with 2.40 ± 0.12 fold (*t* = 0.09) and in systemic leaf with 1.8 ± 0.06 fold (*t* = 0.01) was seen in adjacent leaves of aphid-infested plants. The levels of *AOS* mRNA in the systemic leaves of infested and uninfested plants were not significantly different. Similarly, in aphid-fed local leaves, relative expression of the SA-responsive gene *PR-5* with 4.41 ± 0.53 fold (*t* = 0.01) and in systemic leaf with 1.4 ± 0.25 fold (*t* = 0.20) was significantly upregulated, although no similar alterations were observed in systemic leaves. This research shows that when *A. gossypii* feeds on *S. lycopersicum*, the plant mounts a local defense response.

### 2.2. Assembly of A. gossypii Saliva

**Figure 2:**
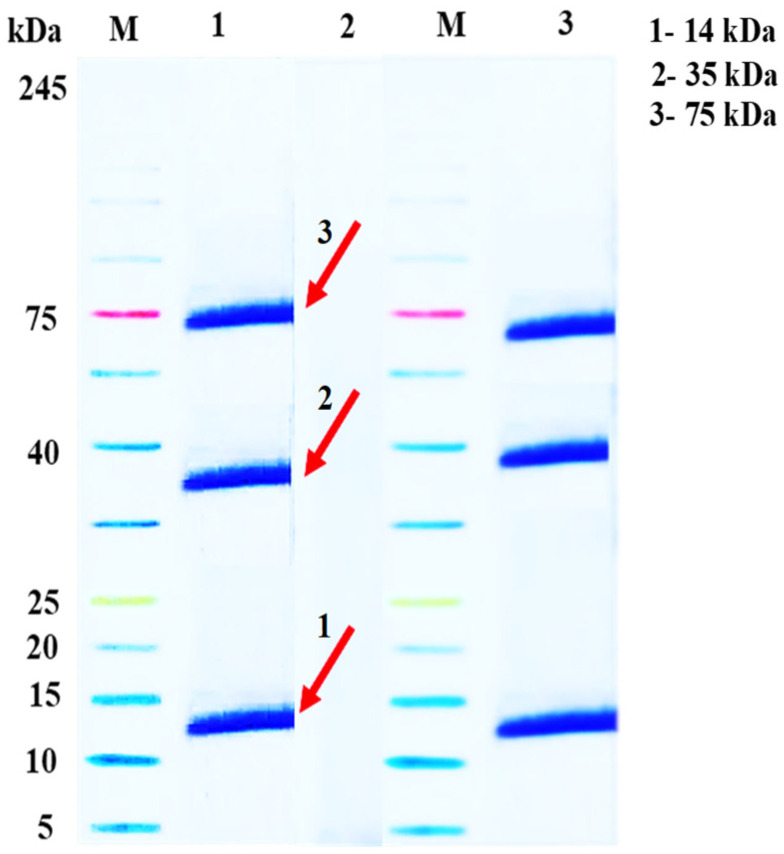
Tricine SDS-PAGE resolving gel of salivary proteins of *A. gossypii*. *Aphis gossypii* salivary protein bands was accomplished using a 12% SDS-PAGE re-solving gel, and a GenStar M223 protein marker (~5–245 kDa) was used for separation and subsequent staining M: protein ladder/protein molecular mass marker; 1: infiltrated saliva sample; 2: Aphis gossypii concentrated saliva sample (without aphids feeding) control sample; 3: infiltrated saliva sample. The red arrows indicate the size of protein band at 14, 35, and 75 kDa respectively.

The detection of *A. gossypii* salivary protein bands was accomplished using a 12% SDS-PAGE resolving gel, and a GenStar M223 protein marker (~5–245 kDa) was used for separation and subsequent staining. The outcomes depicted in Figure 2 indicate a clear and distinct staining of the protein bands, i.e., M: protein ladder/protein molecular mass marker; 1: infiltrated saliva sample; 2: *A. gossypii* concentrated saliva sample control sample; 3: infiltrated saliva samples which were primarily observed to be localized at 14-kDa range, as well as at the 35 kDa and 75 kDa range.

### 2.3. Defense-Related Gene Expression after A. gossypii Saliva Invasion

**Figure 3:**
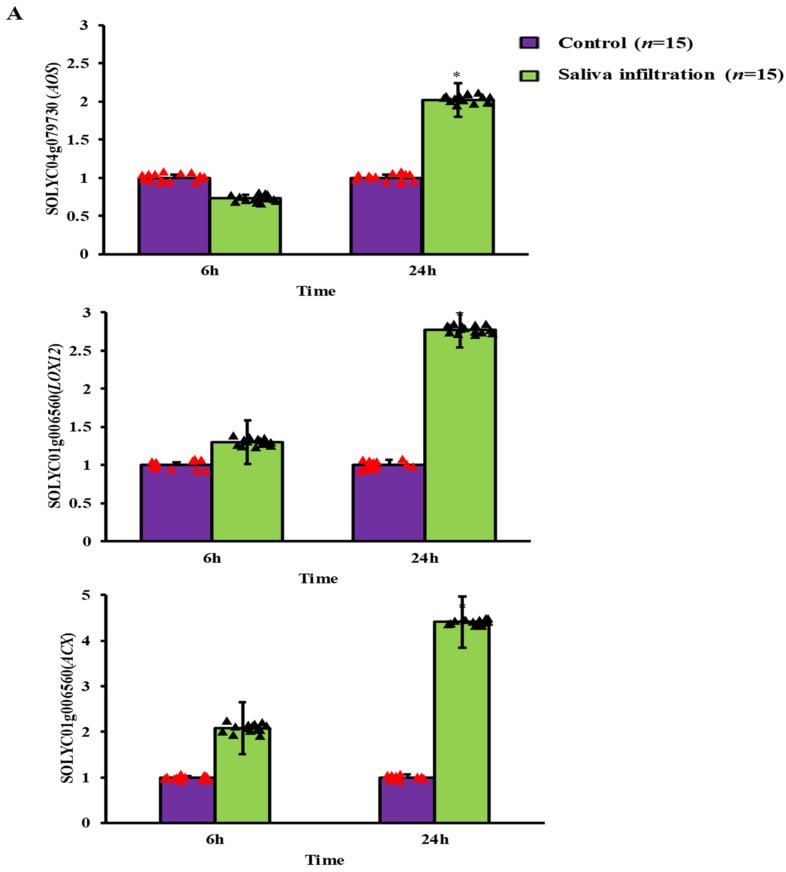

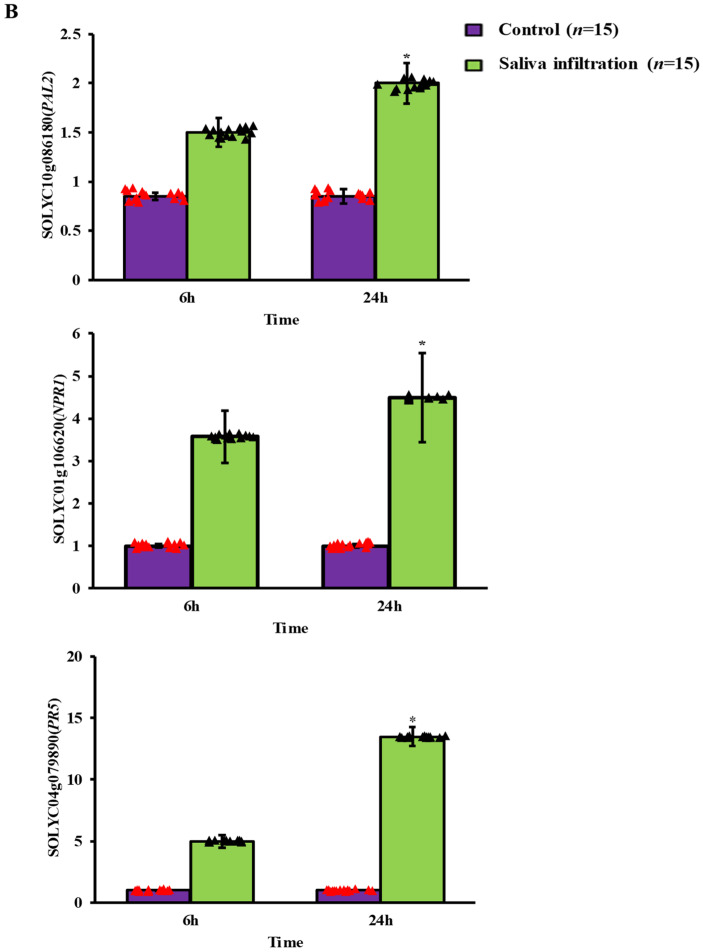
Genes implicated in the JA (**A**) and SA (**B**) defensive responses are expressed in relation to one another in *S. lycopersicum* leaves following saliva penetration. The results are shown as mean ± SE. Purple bars represent the control group, and green bars show *A. gossypii* saliva penetration. Significant differences between treatments are indicated by a “*” (*p* < 0.05, *t*-test, *n* = 15).

We found that the defense pathways against salicylic acid (SA) and jasmonic acid (JA) were expressed differently in *S. lycopersicum* leaves Figure 3A,B. After 6 h of infiltration, there was no discernible change between the treatment and control conditions for any of the three JA-responsive genes. The enzyme responsible for SA synthesis, *PAL2*, for 6 h with 1.5 ± 0.04 fold (*t* = 0.01) and 24 h with 2.0 ± 0.07 fold (*t* = 0.01), and the downstream signaling molecule *PR-5*, for 6 h with 5.06 ± 0.04 fold (*t* = 0.01) and 24 h with 13.5 ± 0.04 fold (*t* = 0.02), and *NPR1* for 6 h with 3.57 ± 0.62 fold (*t* = 0.01) and 24 h with 4.5 ± 1.05 fold (*t* = 0.01) were significantly up regulated, however. After being exposed to saliva for 24 h, the relative expression of the JA defense-related gene *AOS* for 6 h with 0.73 ± 0.05 fold (*t* = 0.01) and 24 h with 2.02 ± 0.22 fold (*t* = 0.02) was significantly higher than in the control group. As compared to the control treatment, the expression level of *LOX12* for 6 h with 1.3 ± 0.29 fold (*t* = 0.31) and 24 h with 2.77 ± 0.23-fold (*t* = 0.01) and *ACX* for 6 h with 2.08 ± 0.57-fold (*t* = 0.07) and 24 h with 4.41 ± 0.56 fold (*t* = 0.01) was likewise up regulated by, showing a significant increase. Additionally, saliva treatment significantly increased the mRNA levels of the enzymes *PAL2* and *NPR1*, which are implicated in the SA production pathway. After saliva treatment, there was a substantial up-regulation in the expression of the SA signaling marker protein *PR-5* compared to the control.

### 2.4. Performance of the A. gossypii

**Figure 4:**
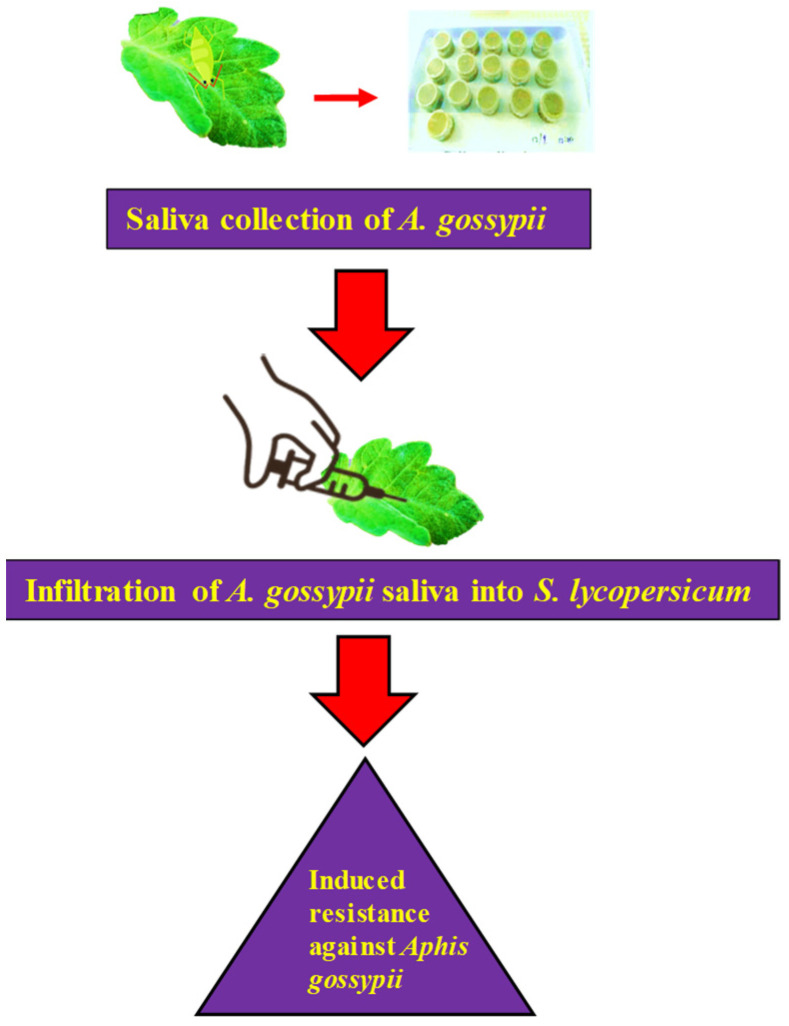
The saliva infiltration setup and procedure, step 1 indicate the initial process of collection and when it was infiltrated into *S. lycopersicum* leaves which caused the mechanism of induced resistance in tomato against *A. gossypii*.

Table 1, Figure 4 demonstrate that there were significant differences between the saliva-treated groups and the control group in terms of the time required for *A. gossypii* to mature or the average relative growth rate. When fed aphid-infested *S. lycopersicum* leaves, *A. gossypii* significantly reduced its daily nymph production in the first generation, and partially reduced it in the second and third generation of aphids, respectively, on saliva treated as compared to control. The intrinsic population growth rate of *A. gossypii* was dramatically suppressed in *S. lycopersicum* plants treated with aphid saliva compared to controls. After 6 h, a considerably smaller percentage of aphids settled on leaves treated with aphid saliva than on control groups, as determined by a choice test. The number of aphids falling on saliva-treated leaves decreased considerably after 24 and 48 h compared to control leaves (Table 2).

### 2.5. Aphis gossypii Feeding Activity by EPG

The investigation into how saliva-treated leaves affected *A. gossypii* feeding behavior is shown in Table 3. When compared to the control *S. lycopersicum*, it was found that aphids spent less time probing *S. lycopersicum* with saliva infiltration. More than twice as many and long non-probing waveforms (np) were present in saliva-infiltrated leaves as compared to control leaves. Additionally, compared to the control group, the saliva treatment group showed a significantly higher frequency and length of C waves. In the saliva-treated group, there were twice as many short probes (C < 3 min) vs. control. Similar to this, *S. lycopersicum* that had been affected with saliva had a significantly larger number of pd waveforms [21,22]. In comparison to the beginning of the EPG recording, aphids demonstrated a greater latency (10 min) after saliva entry in obtaining the first sustained E2 wave. In contrast, the control group had considerably longer E2 waves than the treatment group, both overall and at their maximum duration. Surprisingly, the presence of F waves was nonexistent in the control group, but their quantity and duration significantly increased following saliva treatment.

## 3. Discussion

In response to aphid feeding, plants activate multiple local and systemic defensive mechanisms [23]. We conducted both local and systemic induction assays on *S. lyscopersicum* leaves following *A. gossypii* feeding, and found that the increased expression of JA- and SA-responsive genes was localized to certain leaf areas. Previous studies showed that *M. persicae* infestation generates considerable transcription of the *PR-1* promoters at nurturing places and local aphid confrontation in *Arabidopsis*, and our results are consistent with those studies [24]. Furthermore, following 24 h of saliva infiltration, the expression levels of JA defense-related genes *AOS* and *LOX* dramatically increased, despite the fact that the fold changes of induction were moderate. All three genes in the SA pathway were significantly up-regulated after only 6 h of infiltration. According to these results, the JA defense response triggered by *A. gossypii* saliva infiltration is rather weak, but the SA defense response is strongly triggered. The SA response in tomato appears to be more vulnerable to *A. gossypii* saliva than the JA reaction. Similar defense responses are triggered in *S. lycopersicum* by *A. gossypii* saliva infiltration as are triggered by direct aphid feeding. *Arabidopsis* leaves infected with *M. persicae* showed elevated expression of *PR-5* and *NPR1*, which are implicated in the SA pathway, as well as *AOS* and *LOX12*, which are involved in JA signaling. Among these routes, the SA signaling pathway was more activated than the JA signaling pathway when *M. persicae* was fed [9, 25]. Although syringe saliva infiltration differs from natural aphid feeding behavior, it is a reliable method for elucidating the functions of salivary components in inducing plant resistance. This approach has also been used successfully in research on *Bemisia tabaci* [25].

According to our findings, aphid feeding and saliva infiltration activate a powerful SA-signaling defense system while only slightly activating the JA-mediated defense route. In other plant–hemipteran interactions, antagonistic crosstalk between these two signal-ing channels has been seen and is interpreted as a conserved adaptive mechanism, which may explain the present finding [26], The JA-responsive response is robust and provides substantial protection from phloem-feeding insects. When JA or MeJA was sprayed on tomato plants, it induced the production of defense proteins including PIs and PPO, which in turn reduced the growth and number of *M. persicae* [27]. Similarly, Ellis et al. (2002) [28] found that *M. persicae* population growth was considerably inhibited in *Arabidopsis* mutants with expression of JA-responsive genes in a constitutive manner. In contrast, the JA-insensitive *Arabidopsis* mutant coi1 displayed a faster rise in aphid population, emphasizing the role of JA in *M. persicae* resistance. The “decoy hypothesis” postulates that hemipterans may employ antagonistic hormonal crosstalk between SA and JA to prevent a potentially more destructive and effective JA response. This hypothesis is based on the idea that hemipterans may use the crosstalk between these two hormones [29]. In addition, it has been proven that the silver leaf *B. tabaci* can alter plant signaling in such a way as to reduce the effectiveness of JA-regulated responses, hence boosting plant vulnerability as well as its own performance [30].

A thorough EPG investigation demonstrated that *S. lycopersicum* treatment with saliva had a substantial impact on *A. gossypii* feeding behavior [31]. In the presence of saliva, the quantity of np waveforms rose, and their duration also increased; this suggests that the aphid stylet did not make electrical contact with the surface of the plant and continued to be inactive. Furthermore, our research found that wingless *A. gossypii* preferred landing on *S. lycopersicum* leaves that were not treated with saliva, as evidenced by a choice test. These findings provided compelling evidence that saliva infiltration resulted in the production of indirect defenses in *S. lycopersicum*, resulting in aphid repellence. Induced plant volatiles have been found as significant participants in the process of warding off aphids and luring their natural predators, so serving as an indirect defense mechanism for plants [32]. Notably, when soybean aphids infested the plants, the production of D-limonene, methyl salicylate, and (E,E)-α-farnesene rose dramatically, attracting the aphid predator, the seven-spot ladybird *Coccinella septempunctata*, mainly through the volatile methyl salicylate [33]. Similar to this, when *Rhopalosiphum padi* infested wheat seedlings, volatile chemicals such as 6-methyl-5-hepten-2-one and methyl salicylate were produced, which inhibited the attraction of aphids to the *S. lycopersicum* plants [34]. In addition, the findings suggested that particular enzymes present in aphid saliva may play an important role in the activation of indirect defensive mechanisms in *S. lycopersicum,* e.g., *S. lycopersicum* leaves that were treated with a pectinase solution attracted the female parasitoid *Lysiphlebus testaceipes*, which resulted in higher rates of aphid parasitism than *S. lycopersicum* leaves that were not treated with pectinase [35].

Feeding on *S. lycopersicum* coated with aphid saliva also changed the insects’ penetrative behavior. In the aphid species *A. gossypii*, an increase in the frequency of C and pd and short probe waveforms coincided with a general lengthening of the extracellular stylet pathway. Aphids exposed to saliva took longer to generate a continuous E2 wave during their penetration phase. According to these findings, resistance factors were boosted in *S. lycopersicum* mesophyll cells as a result of aphid saliva infiltration. Passive ingestion (E2 wave) by *A. gossypii* was reduced while aphids fed on treated *S. lycopersicum*; it showed that they had big difficulty sustaining their nutrition uptake from the phloem over time. Since the aphids had trouble feeding on *S. lycopersicum* infused with saliva, the existence of inducement susceptibility inside the sieve elements was demonstrated by the development of F waves that are specific to the saliva-treated group. Aphid infestations, it should be noted, can cause callose deposition both at the feeding site as well as inside the phloem sieve elements. According to a group of researchers [36], the aphids’ ability to constantly feed on phloem sap is disrupted by an increase in callose deposition, which clogs the sieve components. Antibiotic secondary metabolites like lectins, phenolic compounds, and reactive oxygen species (*ROS*) are produced due to the accumulation of aphids, mesophyll cells, and phloem sap, where they exert a suppressive effect on aphid performance [37]. Based on these findings, we hypothesized that the subsequent decrease in aphid feeding activity was caused by *S. lycopersicum* induced resistance mechanisms. This could lead to a decrease in the intrinsic growth rate and the mean reproduction rate of the *A. gossypii* population.

Using a transient transformation system mediated by *Agrobacterium tumefaciens*, researchers have been able to successfully overexpress aphid saliva proteins in plants like *Arabidopsis*, tobacco, and tomato to study the roles of putative salivary elicitors and effectors in aphid–plant interactions [38,39]. Agrobacterium-mediated transformation in cereals is not particularly effective, thus there has not been much work carried out to identify and characterize possible elicitors in *S. lycopersicum*–aphid interactions. Sending proteins into cereal plant cells via a plant pathogen type III secretion system (T3SS) has been shown by wheat or barley with the ability to deliver fungal effectors effectively [40]. This data suggests that the EtHAn can be used effectively as a grain additive. In addition, Upadhyaya et al. (2014) [41] used the expression vector pNR526 to transport *P. fluorescens* EtHAn and identified an effector from the stem rust fungus *Puccinia graminis* f. sp. *tritici* that induced a hypersensitive reaction (HR) in wheat based on the host’s genotype. Delivery of the potential effector PSTha5a23 from the fungus *Puccinia striiformis* f. sp. *Tritici*, by utilizing the pEDV6 expression vector in conjunction with the *P. fluorescens* EtHAn delivery system, inhibited callose deposition connected to pattern-triggered immunity in wheat, as reported by Cheng et al. (2016) [42].

## 4. Materials and Methods

### 4.1. Rearing of Insects and Prepration of Plant Colonies

This study utilized *S. lycopersicum* (Gaili-angmaofen802F1, Jiaxin Seed Limited Company, Jiaxing, China). To ensure surface sterilization, the seeds were immersed in a 0.5% sodium hypochlorite solution (Amresco, OH, USA) for 30 min, followed by three washes with distilled water. After sterilizing the seeds, they were placed on sterilized petri dishes and germinated in filtered water at a temperature of 23 ± 1 °C for 5–6 days. The water in the petri dishes was changed daily. We carefully moved healthy seedlings of equal size into plastic pots with organic soil, which consisted of a mixture of peat and vermiculite in a ratio of 3:1. The seedlings were reared in a climate chamber under controlled conditions until they reached the two-leaf stage. The climate chamber provided a light-dark cycle of 16 h of light and 8 h of darkness (L:D = 16 h:8 h) with a temperature of 20 ± 1 °C. A solitary *A. gossypii* aphid was collected from a wheat field in Guiyang City, China, and used to establish a clone. For three years, the aphid clone was reared on *S. lycopersicum* plants of the Gaili-angmaofen802F1 variety, with approximately 22–25 generations per year. The aphids were reared indoors under controlled conditions with a temperature of 22 ± 1 °C, relative humidity ranging from 40% to 60%, and a photoperiod of 16 h of light and 8 h of darkness (L:D = 16 h:8 h).

### 4.2. Aphid Infestation Treatments

At the two-leaf development stage, 20 apterous adult *A. gossypii* aphids were introduced onto the first (older) leaf of *S. lycopersicum* plants. To prevent aphid escape, we placed the infested leaf inside a plastic ecological cage (2.7 × 2.7 × 2.7 cm). The edges of the cage were covered with a sponge to prevent any mechanical damage to the leaf. This leaf, where aphids fed, was referred to as the “local leaf” assemblage. The second leaf on the same plant was referred to as the “systemic leaf” and was caged as well, but it was free of aphids. For the control group, no aphids were present in the cages that housed both leaves. Each potted plant consisted of a single *S. lycopersicum* plant and was placed in a climate incubator with a temperature set at 20 ± 1 °C and a photoperiod of 16 h of light followed by 8 h of darkness (L:D). Thirty minutes later, all aphids had settled and begun feeding at this moment, which was recorded as 0 h. All aphids were carefully removed after 48 h of continuous feeding, and leaf samples were obtained. The study employed a CRD randomized statistical design with 15 replicates per treatment; the experiment was repeated twice individually.

### 4.3. Collection of Aphid Saliva

Chemically defined diets for *A. gossypii* were formulated as previously described by Ashford et al. (2000) [43] and sterilized with 0.22 μm Millipore membrane filters (Merck Millipore, Germany). Then, 1 mL of artificial diet was sandwiched between two layers of Parafilm membrane (Bemis, WI, USA) stretched across a PVC tube, 27 mm in diameter and 40 mm high, under sterile conditions. The Parafilm was sterilized and exposed to UV light for a minimum of 1 h before use. Approximately 200 *A. gossypii* of different instars were carefully collected from *S. lycopersicum* plants and starved for 2 h, then transferred to the PVC tubes to feed on the artificial diet for 48 h in an environmental chamber (20 ± 1 °C, L:D = 16 h:8 h); tubes with the same volume of artificial diet but without aphids feeding were used as a control. The secreted saliva was collected from a total of 100 mL of diet (approximately 25,000 aphids) and stored at −80 °C until use. The salivary sample was concentrated to a volume of 2 mL using a Vivaspin 20 centrifuge concentrator (Sartorius, Gottingen, Germany) with a 3000 Da molecular weight cut-off PES membrane at 4 °C, 15,000× *g* for at least 1 h. Ten microliters of concentrated saliva sample or 5 μL of protein ladder (PageRulerTM Unstained Protein Ladder, Thermo Scientific, Waltham, MA, USA) mixed with an equal volume of loading buffer were heated in boiling water for 5–10 min then loaded into the wells of the gel. Proteins were separated with 5% stacking and 12% SDS-PAGE resolving gel, and a GenStar M223 protein marker (~5–245 kDa) was used for separation and subsequent staining.

### 4.4. Saliva Infiltration

In order to perform saliva infiltration, we began by diluting 20 µL of concentrated saliva or a control sample with distilled water to a final amount of 200 µL. This brought the total volume of both samples to the same level. Subsequently, using this diluted solution, at the two-leaf stage, the first leaf of a *S. lycopersicum* plant was injected with a 1 mL needleless syringe [44]. The control groups’ leaves were infiltrated with the same volume of the control sample as the other groups’ leaves. All the plants were then cultivated under identical environmental conditions as previously described above to enable further investigations. After 6 h and 24 h of infiltration, the infiltrated leaves were collected for subsequent analysis.

### 4.5. Isolation of Total RNA and Synthesis of cDNA

Using sanitized scissors, leaf samples were collected and promptly frozen in liquid nitrogen. The samples were then frozen at −70 °C until use. The total RNA was extracted using TRIzol^®^ Reagent (Invitrogen, Carlsbad, CA, USA) according to the manufacturer’s instructions. The quality and quantity of RNA were evaluated using NanoDrop™ 2000 Spectrophotometers (Thermo Scientific, Waltham, MA USA). Using the Transcript One-Step gDNA Removal and cDNA Synthesis Super Mix kit (TransGen Biotech, Beijing, China), 1 μg of the isolated RNA was reverse transcribed into complementary DNA (cDNA). cDNA was synthesized according to the manufacturer’s guidelines. The resulting cDNA templates were stored at −20 °C until they were used for real-time quantitative polymerase chain reaction (RT-qPCR) analysis.

### 4.6. RT-qPCR Analysis

Reverse transcription quantitative polymerase chain reaction (RT-qPCR) was used to evaluate the relative expression levels of genes implicated in the jasmonic acid (JA) and salicylic acid (SA) defense signaling pathways in *S. lycopersicum*. The JA-responsive pathway target genes included lipoxygenase SOLYC01g006560 (*LOX12*), allene oxide synthase SOLYC04g079730 (*AOS*), and acyl-coenzyme A oxidase SOLYC04g054890 (*ACX*), all of which are known to be involved in JA biosynthesis [45]. For the SA-responsive pathway, the genes tested were phenylalanine ammonia-lyase SOLYC10g086180 (*PAL2*), which are enzymes involved in SA synthesis, and pathogenesis-related protein 1, SOLYC01g106620 (*NPR-1*), and SOLYC04g079890 (*PR5*), an SA-induced marker protein [46]. The housekeeping gene actin was used as an internal control, and its primer sequences were designed according to Liu et al. [45,46]. The primers for RT-qPCR were designed using Primer Premier 5.0, and their sequences are provided in Table 4. The RT-qPCR analysis was carried out on an ABI 7500 Real-Time PCR System (Applied Biosystems, Carlsbad, CA, USA). The cDNA samples were subsequently diluted 10-fold and utilized as templates in a reaction system that contained 20 µL. Each reaction used 0.4 µL of 50× ROX Reference Dye II (Tli RNaseH Plus, Takara, Dalian, China),10 µL of 2× SYBR premix Ex Taq^TM^ (Tli RNaseH Plus, Takara, Dalian, China), 2 L of cDNA, and 0.5 µL each of 10 µmol L^−1^ forward and reverse primers. The total volume of each reaction was 2 L. The following describes the conditions that were used during the amplification process: initial denaturation at 95 °C for 30 s, followed by 40 cycles of denaturation at 95 °C for 30 s, and annealing/extension at 60 °C for 40 s. In RT-qPCR, with 15 replicates per treatment, the experiment was repeated twice individually.

### 4.7. Aphid Bioassay

#### 4.7.1. *Aphis gossypii* Preference Assay

The goal of this experiment was to assess the host selection of *A. gossypii*. After an incubation period of 24 h with saliva, *S. lycopersicum* plants were laid out in a horizontal position on a level surface. A clear plastic column (24 cm wide, 177 cm tall) with holes in opposing sides was used to gently insert leaves of the same height (5 cm) with two different treatments, i.e., saliva treated and control. Using a 2.0 mL centrifuge tube, we collected 30 wingless *A. gossypii*, and then the thirty wingless *A. gossypii* adults were released into the middle of the plastic column device. The number of *A. gossypii* on each leaf was recorded at 6, 24, and 48 h after release and all trials were conducted in a climate-controlled room. The photoperiod of 16 h of light was followed by 8 h of darkness, and the chamber was kept at a constant temperature of 20 ± 1 °C with a relative humidity of 40–60%. The study employed a CRD randomized statistical design. Seedlings from each plant were separated using transparent air-permeable cages. The experiment was carried out 20 times independently. After 6, 24, 48 h, the *A. gossypii* on each seedling were examined and the number of *A. gossypii* on each seedling was recorded.

#### 4.7.2. Intrinsic Population Growth Rate of *A. gossypii*

This experiment aimed to examine if nourishing saliva-treated or control seedlings increased *A. gossypii* intrinsic development. *Solanum lycopersicum* seedlings were treated the same way as described previously, i.e., the initial leaves of *S. lycopersicum* plants at the two-leaf stage were infiltrated with the secretions of *A. gossypii*. After 24 h, a newly-hatched aphid was transferred to a leaf treated with saliva. After one day, the seeds were treated with saliva solution. A glass tube wrapped with cotton gauze segregated sprouting, and *A. gossypii* mortality on the leaves was confined in a plastic ecological cage (Cangzhou Hengyun Plastic Industry, Cangzhou, China) (2.7 × 2.7 × 2.7). The ecological cage’s periphery was sponge-coated to eliminate mechanical damage to the leaf. The *A. gossypii* instars 1st–4th were tested for larval development for 12 h. To avoid overcrowding, newly molted larvae were tallied twice daily to govern the overall aggregate of period and offspring produced. This was repeated on seeds and plants five days afterwards. The experiment employed 30 replicates of each treatment. The intrinsic rate was determined in the following manner: *r_m_* = 0.738 (ln*Md*)/*Td* [47,48,49], where *Md* is the number of newborn larvae within a development period of *Td*, and *Td* is the time interval between an *A. gossypii* birth and reproduction. With 30 replicates for each treatment, the experiment was repeated twice individually. Every three days, the seedlings of *S. lycopersicum* were exchanged for new seedlings that were given the same kind of treatment. This was done so that the experimental conditions would remain consistent throughout.

### 4.8. EPG Monitoring of A. gossypii Eating Activity

We monitored *A. gossypii* as it munched on *S. lycopersicum* leaves using (EPG) on a Giga-8d system. Apterous adult *A. gossypii* were brush-collected off *S. lycopersicum* plants and starved for 30 min. After that, we used silver glue diluted in water to attach several centimeters of 18-gauge gold wire to the aphids’ bellies. Successful electrical access was achieved by inserting a plant electrode into the soil of a container. The probe’s input (a BNC connector) was then carefully connected to the insect electrode, wire and all. Every day, from 10:00 to 16:00, 6 h of continuous EPG recordings were made. Each aphid and plant were only used once in the trials, which ensured that the results were accurate and reliable. All experiments were conducted in a Faraday cage at a constant temperature of 20 ± 1 °C. We used the Stylet^+^d program to visually depict and manually name the various feeding waves. Sarria et al. (2009) [50] detailed a methodology for identifying the properties of aphid feeding waves, which were then used in the present investigation. The mean of twenty replications was subjected to statistical calculation (*n* = 20). With 20 replicates per treatment, the experiment was repeated twice individually.

### 4.9. Data Analysis

In the data analysis section, the statistical software SPSS 17.0 was used for analyzing the data. Before conducting the analysis, the arcsine-square-root transformation was applied to the percentages of *A. gossypii* that were found to have fallen on plant leaves during the choice test. The Student’s *t*-test was used to investigate the differences between groups. For the EPG (electropenetrography) data, a Mann–Whitney U test was used for analysis. For the RT-qPCR (reverse transcription quantitative polymerase chain reaction) analysis, each treatment was performed in triplicate. The differential expression was calculated using the CT (2^−∆∆CT^) method, which is a commonly used method in gene expression analysis [51]. Between the control and treatment conditions, the fold change in the expression of genes implicated in the JA (jasmonic acid), SA (salicylic acid), and signaling defense pathways was determined. The statistical analysis of the fold change data was performed using Student’s *t*-test. A significance level (α) of 0.05 was used to determine statistical significance, which means that *p* values less than 0.05 were considered statistically significant.

## 5. Conclusions

In this investigation, we found that *A. gossypii* saliva significantly affected *S. lycopersicum* leaves, resulting in strong SA defense responses and comparatively modest JA defense responses. The enhanced expression of genes linked to both the SA and JA pathways made this clear. Surprisingly, saliva-infiltrated leaves showed a repelling effect on aphids, impairing feeding behavior, lowering the mean reproduction rate, and generally slowing population development. These results strongly imply that *A. gossypii* salivary components are essential for conferring resistance against aphids in *S. lycopersicum* plants. Our ongoing research will mainly concentrate on creating a workable mechanism for directly delivering salivary proteins from *A. gossypii* to *S. lycopersicum* plants using a T3SS (Type III secretion system). With this strategy, we hope to pinpoint the precise elicitors that cause resistance induction in *S. lycopersicum*. This research project shows great potential for elucidating the mechanisms underpinning interactions between *S. lycopersicum* and aphids and ultimately opening the door for practical methods to strengthen *S. lycopersicum* resistance to these destructive pests.

## Figures and Tables

**Table 1:** *Aphis gossypii* development duration, everyday reproduction proportion, intrinsic rate of surge, and mean relative growth rate following feeding on saliva-infiltrated *S. lycopersicum* leaves and a control diet. The data were presented as mean ± SE. “*” indicates that there were significant differences between treatments (*p* < 0.05, *t*-test).

Generations of *A. gossypii*	Td (Day) (*n* = 30 for Each Aphid Generation)		No. of Nymphs per Day (*n* = 30 for Each Aphid Generation)		*r_m_* (*n* = 30 for Each Aphid Generation)	
	Saliva Infiltration	Control	Saliva Infiltration	Control	Saliva Infiltration	Control
1st	8.63 ± 0.02	8.54 ± 0.01	3.73 ± 0.01	4.10 ± 0.02	0.32 ± 0.001	0.36 ± 0.001
2nd	8.54 ± 0.02	7.44 ± 0.03	3.34 ± 0.012	3.53 ± 0.01	0.29 ± 0.01 *	0.35 ± 0.01 *
3rd	8.89 ± 0.03	8.74 ± 0.007	2.42 ± 0.02	2.43 ± 0.018	0.19 ± 0.002 *	0.21 ± 0.01 *

**Table 2:** *Aphis gossypii* colonization on *S. lycopersicum* saliva-leaves and control leaves, mean (±SE) the study used randomized statistical design CRD, Data were compared using one way Anova and least significant difference (LSD) (*p* = 0.05, *n* = 20) between groups is shown by a and b which are significant differences among treatments and control “*”.

Time in (h)	Percentage of Aphids	
	Saliva Infiltration (*n* = 20 for Each Time Period)	Control (*n* = 20 for Each Time Period)
6 h	25.21 ± 0.01 ^b^	45.17 ± 0.02 *^a^
24 h	44.10 ± 0.02 *^b^	54.10 ± 0.05 ^a^
48 h	48.13 ± 0.01 ^b^	63.12 ± 0.04 ^a^

**Table 3:** *Aphis gossypii* EPG parameters on saliva infiltrated and controlled *S. lycopersicum* leaves (mean ± SD). The data was compared using a *t*-test with two-tailed significance (*p* = 0.05, *n* = 20). The significant differences, Asterisks indicate significant differences between Saliva-Infiltrated treatment and control with the same parameters of * (*p* = 0.05).

Parameters of the EPG	Saliva-Infiltrated (*n* = 20 for Each Parameter)	Control (*n* = 20 for Each Parameter)
Total amount of time spent probing (h)	4.22 ± 0.13	5.19 ± 0.07 *
Number of C	18.12 ± 0.01 *	10.94 ± 0.15
Number of short probes (C < 3 min)	8.41 ± 0.02 *	4.15 ± 0.11
Duration of non-probe period before the 1st E (h)	2.71 ± 0.02	2.62 ± 0.05
Mean duration of pd (s)	7.52 ± 0.03	7.81 ± 0.02
Number of pd	131.21 ± 0.11 *	91.25 ± 0.05
Number of E1	7.51 ± 0.10	6.41 ± 0.02
Mean duration of E1 (min)	11.15 ± 1.01	9.81 ± 0.22
Number of E2	4.61 ± 0.03	5.24 ± 0.11
Mean duration of E2 (h)	0.16 ± 0.02	1.51 ± 0.02
Number of G	0.16 ± 0.01	0.72 ± 0.02
Mean Duration of G (min)	12.13 ± 0.02	11.35 ± 0.01
Number of F	0.21 ± 0.02 *	0.91 ± 0.02
Mean duration of F (min)	3.11 ± 0.03 *	2.12 ± 0.03

**Table 4:** Primers for all tested genes.

Test Genes	Pathways	Forward Sequence (5′……3′)	Reverse Sequence (5′……3′)
SOLYC04g079730 (*AOS*)	JA	GCTACAATTCCCCTCGCATA	ACAGGTGGTGATGACGATGA
SOLYC01g006560 (*LOX12*)	JA	AGAGGCGGTTCAACAAAAGA	CAATCGCCAAAGGTCTCAAT
SOLYC04g054890 (*ACX*)	JA	GCAGCCTGCCTACAATTAGC	GCAAAATGGAATGCGTAGGT
SOLYC10g086180 (*PAL2*)	SA	GGATCCTTTGCAGAAACCAA	GCCACCATGTAAAGCCTTGT
SOLYC01g106620 (*NPR1*)	SA	TACTCAGGTGGTGTGGCGTA	TCAAAAGCCGGTTGATTTTC
SOLYC04g079890 *(PR5*)	SA	CTGGGCAATGAACAGGATTT	AAGCACTTTTGCAAGCCACT
*Actin*		GTGACGGGTGACGGAGAATT	GACACTAATGCGCCCGGTAT

## Data Availability

The required data set is already available in manuscript file; other data sets generated during the study are available upon request from corresponding author.
